# Mapping of the Language Network With Deep Learning

**DOI:** 10.3389/fneur.2020.00819

**Published:** 2020-08-05

**Authors:** Patrick Luckett, John J. Lee, Ki Yun Park, Donna Dierker, Andy G. S. Daniel, Benjamin A. Seitzman, Carl D. Hacker, Beau M. Ances, Eric C. Leuthardt, Abraham Z. Snyder, Joshua S. Shimony

**Affiliations:** ^1^Department of Neurology, Washington University School of Medicine, St. Louis, MO, United States; ^2^Mallinckrodt Institute of Radiology, Washington University School of Medicine, St. Louis, MO, United States; ^3^Department of Biomedical Engineering, Washington University, St. Louis, MO, United States; ^4^Department of Neurosurgery, Washington University School of Medicine, St. Louis, MO, United States

**Keywords:** functional MRI, language, deep learning, resting state network, convolutional neural network

## Abstract

**Background:** Pre-surgical functional localization of eloquent cortex with task-based functional MRI (T-fMRI) is part of the current standard of care prior to resection of brain tumors. Resting state fMRI (RS-fMRI) is an alternative method currently under investigation. Here, we compare group level language localization using T-fMRI vs. RS-fMRI analyzed with 3D deep convolutional neural networks (3DCNN).

**Methods:** We analyzed data obtained in 35 patients with brain tumors that had both language T-fMRI and RS-MRI scans during pre-surgical evaluation. The T-fMRI data were analyzed using conventional techniques. The language associated resting state network was mapped using a 3DCNN previously trained with data acquired in >2,700 normal subjects. Group level results obtained by both methods were evaluated using receiver operator characteristic analysis of probability maps of language associated regions, taking as ground truth meta-analytic maps of language T-fMRI responses generated on the Neurosynth platform.

**Results:** Both fMRI methods localized major components of the language system (areas of Broca and Wernicke). Word-stem completion T-fMRI strongly activated Broca's area but also several task-general areas not specific to language. RS-fMRI provided a more specific representation of the language system.

**Conclusion:** 3DCNN was able to accurately localize the language network. Additionally, 3DCNN performance was remarkably tolerant of a limited quantity of RS-fMRI data.

## Introduction

In treating brain tumors, the neurosurgeon must balance the benefit of maximal tumor resection against the risk of a functional impairment consequent to more aggressive approaches. These two factors, maximal resection and functional preservation, are often cited in the surgical literature as predictors of long term survival (1–3). Thus, preoperative and intraoperative functional localization is critical to optimizing these often conflicting priorities. Functional MRI (fMRI) has been used as an adjunct measure for preoperative mapping of eloquent cortex and intraoperative navigation (4). The gold standard, however, for defining eloquent cortex is intraoperative mapping using electrical cortical stimulation mapping (5). Since electrocortical stimulation carries clinical risk (6), preoperative mapping to optimize the intraoperative surgical approach is an effective means of preserving function.

fMRI detects changes in the blood oxygen level dependent (BOLD) signal that reflect the neurovascular response to neural activity. In conventional fMRI, function is localized by presenting stimuli or imposing tasks (such as finger tapping or object naming) (7). More recently, resting state fMRI (RS-fMRI), i.e., fMRI obtained in the absence of stimuli or tasks, has been used to map the brain's functional organization (8). RS-fMRI data is simpler to acquire and does not require patient cooperation (important in children and neurologically impaired patients). Thus, RS-fMRI has opened new opportunities for clinical applications, such as pre-surgical planning (9–11).

Multiple techniques have been used to map the representation of function using RS-fMRI data. These techniques include independent component analysis (12), seed-based correlation (13), and machine learning methods such as the multi-layer perceptron (MLP) (14). With typical fMRI acquisition times, these methods are signal to noise limited and have limited sensitivity and specificity. More recently, advances in deep convolutional neural networks (CNN) have revolutionized image classification and segmentation problems (15, 16). It is thus logical to apply CNN methodology to the problem of mapping resting state networks.

In this study, we apply a CNN to RS-fMRI data for the localization of the language system in a cohort of patients with brain tumors and compare these results to the localization estimated in the same patients with task-based fMRI (T-fMRI). We evaluated T-fMRI vs. RS-fMRI as methods for pre-surgical mapping of the representation of language. We used aggregated language T-fMRI data in the Neurosynth platform (www.neurosynth.org) (17) to define language localization ground truth. Our hypothesis is that the current study will provide preliminary data for the utility of CNN methods for the localization of the language system. We further wish to characterize differences in language localization between the CNN and T-fMRI methods.

## Materials and Methods

### Patients

Patients were retrospectively recruited from the Neurosurgery brain tumor service at the Washington University School of Medicine in Saint Louis (WUSM), initially as part of an NIH-funded tumor data base grant (CONDR NIH 5R01NS066905). This patient cohort was used in a prior study targeting non-invasive localization of sensorimotor cortex (18). The following inclusion criteria were used: new diagnosis of primary brain tumor; age above 18 years; clinical need for an MRI scan including fMRI for presurgical planning as determined by the treating neurosurgeon. Additionally, we required that the patients have both a language task (word-stem completion) T-fMRI and RS-fMRI. Exclusion criteria included: prior surgery for brain tumor, inability to have an MRI scan, or a patient referred from an outside institute with an MRI scan not performed at WUSM. Our cohort include *N* = 35 patients (male/female 23/12) with a mean age of 44.8 years (23–71 years range). The mean preoperative enhancing tumor volume was 43.8 mL (range: 1.4–207 mL); 28 patients had a left-hemisphere tumor; pathology was most often oligoastrocytoma (11 cases) and glioblastoma (10 cases). Handedness was recorded in 26 patients. To decrease any uncertainty in regard to laterality we also included the laterality index (LI) for all subjects based on (19). Since the 3 left handed patients had LI > 0, and two of the three patients with LI < 0 were right handed (the handedness on the third was not available) we opted to average language activation in all subjects as a single group. Patient demographics are summarized in Table 1. All aspects of the study were approved by the WUSM Institutional Review Board. Clinical data were acquired during preoperative evaluation and reviewed retrospectively.

**Table 1:** Patient clinical and demographic data.

**Patient ID (*N* = 35)**	**Age (years)**	**Handedness Lat. index**	**Tumor location**	**Tumor size (mL)**	**Tumor pathology**
RS_003	40–45	R/-0.11	Left basal ganglia	8.7	Glioblastoma
			Left temporal lobe	4.8	
RS_004	20–25	R/0.04	Left frontal lobe	56.2	Anaplastic glioma
RS_005	35–40	NA/-0.08	Left frontal lobe	1.2	Anaplastic mixed oligoastrocytoma
			Left frontal lobe	0.2	
RS_006	35–40	NA/0.32	Left inferior frontal lobe	81.1	Anaplastic mixed oligoastrocytoma
RS_007	60–65	R/0.15	Left parieto-occipital	85.1	Glioblastoma,
RS_009	60–65	R/0.10	Left peri-trigonal area	147	Glioblastoma
RS_011	20–25	R/0.67	Left frontotemporal	56.4	Mixed oligoastrocytoma
RS_012	40–45	R/0.36	Left frontal lobe	7.8	Anaplastic oligodendroglioma
RS_014	40–45	R/0.37	Left frontal/insular lobe	69.2	Oligodendroglioma
RS_015	60–65	NA/0.05	Left frontal lobe	34.7	Mixed oligoastrocytoma
RS_016	55–60	NA/0.01	Left insula	15.2	Glioblastoma
RS_017	50–55	R/0.08	Left frontal lobe	64.3	Mixed oligoastrocytoma
RS_018	35–40	R/0.35	Left frontal lobe	13.5	Oligodendroglioma
RS_019	30–35	R/0.25	Right frontoparietal	207	Anaplastic oligodendroglioma
RS_020	50–55	R/0.24	Left temporal lobe	19.9	Glioblastoma
RS_021	25–30	R/0.05	Left frontal lobe	63.3	Mixed oligoastrocytoma
RS_022	65–70	NA/0.02	Right frontal lobe	2.2	Metastatic lung carcinoma
RS_023	50–55	R/0.38	Left parietal/splenium	28.7	Oligodendroglioma
RS_024	55–60	R/0.25	Left frontal lobe	4.7	Anaplastic oligoastrocytoma
RS_027	45–50	L/0.48	Left temporal lobe	24.8	Low-grade diffuse glioma
RS_029	50–55	R/0.11	Left frontal lobe	14.5	Oligodendroglioma
RS_030	70–75	R/-0.14	Right basal ganglia/thalamus	16.6	Glioblastoma
RS_031	50–55	NA/0.53	Left thalamus	5.8	Glioblastoma
RS_032	45–50	R/0.79	Right temporal lobe	5.7	Glioblastoma
RS_033	35–40	R/0.64	Left frontal lobe	185	Mixed oligoastrocytoma
RS_034	55–60	NA/0.22	Left temporal lobe	24.9	Meningioma
RS_035	25–30	R/0.13	Left temporal lobe	10.1	Oligoastrocytoma
RS_039	25–30	L/0.18	Right parietal lobe	32.0	Mixed oligoastrocytoma
RS_040	35–40	R/0.29	Right sylvian fissure	31.5	Ependymoma
RS_041	40–45	NA/0.54	Left frontal lobe	23.3	Mixed oligoastrocytoma
RS_042	60–65	R/0.17	Left parietal lobe	0.7	Glioblastoma
RS_043	30–35	R/0.46	Right temporal lobe	4.0	Low-grade glioneuronal tumor
RS_044	20–25	R/0.40	Left frontal lobe	0.4	Ganglioglioma
RS_045	25–30	L/0.33	Bilateral frontal lobes (left>right)	118	Anaplastic astrocytoma
RS_047	55–60	NA/0.16	Left frontal lobe	66.2	Glioblastoma

*Clinical data for 35 patients with brain tumors (age 44.8 ± 14.0 years; 12 female)*.

### MRI Acquisition

Patients were scanned with either a 3T Trio or Skyra scanner (Siemens, Erlangen, Germany) using a standard clinical presurgical tumor protocol. Anatomical imaging included T1-weighted (T1w) magnetization prepared rapid acquisition gradient echo (MP-RAGE), T2-weighted (T2w) fast spin echo, fluid-attenuated inversion recover (FLAIR), susceptibility-weighted imaging (SWI), and pre/post-contrast T1w fast spin echo in three projections. Additional sequences for presurgical mapping included Diffusion Tensor imaging (DTI) for track tracing, T-fMRI for motor and language localization, and RS-fMRI.

Both the task and resting state fMRI were acquired using echo planar imaging (EPI) (voxel size 3 × 3 × 3 mm; TE = 27 ms; TR = 2.2 s; field of view = 256 mm; flip angle = 90°). The language T-fMRI employed a block design in which patients covertly generated words in response to a visually presented first letter. Five task/rest blocks (10 frames each) were acquired over a total of 90–100 frames (3:40 min total per T-fMRI run). For most subjects, two language task sessions were acquired, and the run with the lowest root-mean-square head motion measure was used in the present analysis. RS-fMRI was acquired in two 160-frame runs (total of 320 frames = 11:44 min).

### Pre-processing

The fMRI data were preprocessed using previously described techniques using locally written software (4dfp.readthedocs.io) (14). Preprocessing was identical for RS-fMRI and for T-fMRI and included compensation for slice dependent time shifts, elimination of systemic odd-even slice intensity differences due to interleaved acquisition, and rigid body correction for head movement within and across runs. Atlas transformation was achieved by composition of affine transforms connecting the fMRI volumes with the T2-weighted and MPRAGE structural images, resulting in a volumetric time series in (3 mm cubic) atlas space. Additional preprocessing included: spatial smoothing (6 mm full width half maximum Gaussian blur in each direction), voxelwise removal of linear trends over each run, and temporal low pass filtering retaining frequencies <0.1 Hz. Spurious variance was reduced by regression of nuisance waveforms derived from head motion correction and extraction of the time series from regions of white matter and CSF. The whole brain (“global”) signal was included as a nuisance regressor (20). Frame censoring was performed to minimize the impact of head motion on the correlation results (21). Thus, frames (volumes) in which the root mean square (evaluated over the whole brain) change in voxel intensity relative to the previous frame exceeded 0.5% (relative to the whole brain mean) were excluded from the functional connectivity computations (22). All fMRI data acquired in each patient were pooled during preprocessing. Thus, the T-fMRI and RS-fMRI data were mutually co-registered in each patient. Additionally, all T-fMRI and RS-fMRI data in all patients were resampled in a standard atlas space. No attempt was made to correct for the mass effect of tumors. To match acquisition durations of RS-fMRI and T-fMRI (11:44 vs. 3:40 min), we selected 100 contiguous frames from pre-processed RS-fMRI data for comparisons with T-fMRI. Additionally, the full quantity of RS-fMRI data was compared to T-fMRI. T-fMRI responses were evaluated using standard general linear model methods using in house software. Activation maps were generated from the T-fMRI as described in Corbetta et al. (23), smoothed with a 10 mm Gaussian filter, and masked to exclude extra-cranial voxels. Neither response clustering nor thresholding was done.

### Deep Learning—Convolutional Neural Networks Processing

#### Training

Normal human resting state fMRI data (*N* = 2,795) were obtained from the Brain Genomics Superstruct Project (GSP) (Harvard University) (24) and ongoing studies at Washington University in St. Louis including the Alzheimer's Disease Research Center (ADRC) (25), the Dominantly Inherited Alzheimer's Network (DIAN) (26), and studies by the Division of Infectious Diseases HIV Program (HIV) (27) (Table 2). Statistical analysis of network FC (evaluated within and across the default mode network, the dorsal attention network, vision network, and deep gray structures) between the different data sets revealed no significant group effect attributable to study. Each subject had ~14 min of resting state fMRI data (TR = 3,000 ms, 3 mm cubic) which was processed using standard methods developed at WUSM (14). Resting state networks (RSNs) were identified using a set of 169 region of interests (ROI) divided into 11 RSNs (14). Multiple (*n* = 268,000) example sets were generated from the data and then divided into a training (*N* = 18,7600) and validation (*N* = 80,400) sets. A 3D convolutional neural network (3DCNN) with a densely connected architecture (15) was trained to classify brain regions as belonging to *a priori* assigned RSNs. The 3DCNN consisted of 49 layers and 3 dense blocks that performed 3 and 5 cubic convolutions. Batch normalization was used within the network to prevent overfitting and improve performance, and average pooling was used for dimensionality reduction. Training was terminated if the accuracy did not improve after 3 validations. The 3DCNN was implemented in Matlab R2019b (www.mathworks.com).

**Table 2:** Studies used to obtain normal training data.

	**GSP**	**ADRC**	**DIAN**	**HIV**
*N*	1,137	1,289	336	775
Age (std)	21.4 (2.4)	68.1 (7.9)	40.9 (10.9)	44.3 (16.3)
Scanner	Trio	Trio/Biograph	Trio/Verio	Trio/Prisma
Voxel Size in cubic mm	3.0	4.0	3.3	4.0
Flip angle in degrees	85	90	80	90
Repetition time (TR) in ms	3,000	2,200	3,000	2,200
Total number of fMRI frames	248	328	140	328

*Refer to text for acronyms and citations*.

#### Testing

For each of 35 tumor patients the T-fMRI results were compared to the 3DCNN results obtained with matched data samples, i.e., 100 contiguous frames of RS-fMRI. Additionally, the 3DCNN analysis was run using all available data (320 RS-fMRI frames per subject). 3DCNN maps representing the probability of language representation were smoothed with stride-1 mode filtering and length-3 box filtering.

### A Priori Defined Language Regions of Interest (ROI)

Language representation in the brain resides primarily on two areas of the left cerebral cortex: Broca's area, located in inferior frontal cortex (roughly, Brodmann areas 44 and 45) and frontal operculum (28), is required for fluid performance of phonemic or semantic tasks (29). Wernicke's area extends over portions of temporal and parietal cortex and is essential for understanding written or spoken language (30). The Broca-Wernicke model embodies core expressive and receptive language functions but omits auxiliary functions such as reading (31–33).

**Figure 1:**
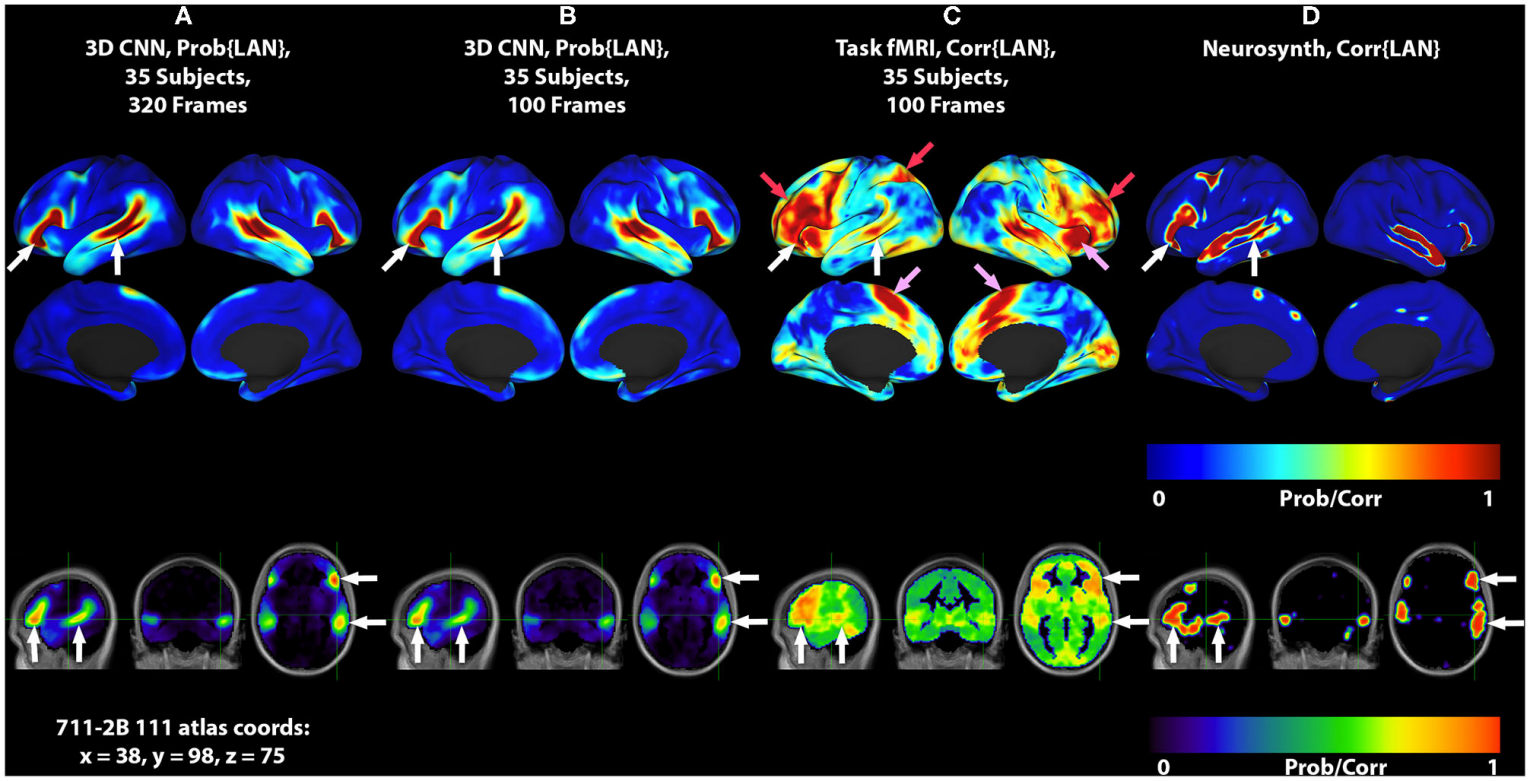
3DCNN maps and T-fMRI responses focusing on language localization. All RS-fMRI and T-fMRI results are averages over 35 patients. Surface plots show probabilities thresholded at *p* > 0.02. Top row shows lateral surface plots; middle row shows medial surfaces; bottom row shows sagittal, coronal, and axial views at coordinate x = 38, y = 98, z = 75 on the 711-2B atlas using radiologic conventions (left body on right image). Columns show: **(A)** 3DCNN language (LAN) map computed using 320 frames per patient (all available RS-fMRI data). **(B)** 3DCNN LAN map computed with only 100 frames per patient. **(C)** Word stem completion T-fMRI responses. **(D)** Neurosynth map derived with the search term, “language comprehension.” White arrows indicate the areas of Broca and Wernicke. Pink arrows indicate task responses in the right anterior insula and dorsal anterior cingulate cortex (core task-control regions). Red arrows indicate task responses in antero-lateral prefrontal cortex and superior parietal lobule (dorsal attention and fronto-pariental control networks).

We defined the ground truth for language representation using T-fMRI responses aggregated by Neurosynth (17). This representation was confined to the left hemisphere to simplify comparison between the Neurosynth regions and those derived in our patients. To define T-fMRI-based language ROIs, we queried Neurosynth using “language comprehension” as a search term, which identified 107 studies (as of November, 2018) contributing coordinates in Talairach atlas space, each coordinate associated with a Z-score corresponding to the null hypothesis of equally likely activation anywhere in the brain. The returned association map (threshold at Z > 3.7 by Neurosynth) was passed through smoothing and clustering operations (see below), ultimately yielding Broca- and Wernicke-like ROIs in volumetric atlas space (Figure 1A).

The following steps were taken to obtain Broca- and Wernicke-like ROIs starting with a “language comprehension” map (units = Z-score) generated by Neurosynth in 2 cubic mm MNI152 atlas space:

1. Gaussian smooth using a kernel of 1 mm full width at half maximum (FWHM) in each cardinal direction.
2. Transform Z-scores >0 to probability maps using the hyperbolic tangent and threshold at Z-score > 3.7.
3. Retain two largest clusters to generate initial estimates of Broca and Wernicke regions in the left hemisphere.
4. Gaussian smooth using a 3 mm FWHM isotropic kernel in each cardinal direction.
5. Resolve overlapping clusters into two disjoint ROIs by assigning multiply labeled voxels to the ROI with the nearest center of mass.

### Image Computation and Visualization Software

fMRI preprocessing, denoising, and computation of RS-fMRI and T-fMRI responses were done using the 4dfp software library (https://readthedocs.org/projects/4dfp/). MATLAB R2019b was used for statistical computations and visualization. Connectome Workbench, version 1.2.3 (www.humanconnectome.org), was used to map volumetric data onto the PALS-B12 mid-thickness surfaces and rendered on the corresponding inflated surface (34).

### Statistical Analysis

RS-fMRI and T-fMRI produce native measurements with distinct statistical properties. Acceptable conventions for image processing, denoising, and significance testing have evolved distinctly for these functional imaging methods. Additionally, highly non-linear deep learning architectures such as 3DCNNs have unknown statistical properties when applied to functional imaging data. To enable meaningful statistical comparisons in our data, we used probabilistic strategies to enhance the detection of the population-invariant language network and reduce the influence of experimental conventions, differential preprocessing and the biological variability arising from the use of normal training data but pathophysiologic testing data. Specifically, we reused common image processing pipelines wherever possible in our analyses. We reduced all method-specific metrics to normalized probability maps. For purposes of group-level inferences, for each patient, all temporal imaging information was contracted into T-fMRI activations or RS-fMRI membership in RSNs. As described in the preprocessing methods, all T-fMRI and RS-fMRI data were co-registered to a standardized atlas. Consequently, spatially distributed measures of task activation or resting state network membership retained co-registration in atlas space. We used arithmetic averages of patient data prior to computing comparative analyses at the group-level. Finally, we used method-dependent thresholds for detection of language networks by analysis of receiver operating characteristics (ROC). ROC computations made exclusive use of the perfcurve method from Matlab. Significance testing at alpha = 0.05 included computation of point-wise confidence intervals on true positive rates by vertical averaging over 101 false positive rate intervals and resampling with 10,000 bootstrap iterations. All other parameterizations of perfcurve were default values.

## Results

### Mean Language Maps

Figure 1 shows group level localizations of the language network. Figures 1A,B are computed from 3DCNN analysis of RS-fMRI, using the full amount of available resting state data (1A), and one third of the available data (1B), comparable to the amount of data available in the T-fMRI. Figure 1C is a group level language map computed from the T-fMRI response to word-stem completion. Figure 1D is derived from the Neurosynth platform (17) using the search term “language comprehension.” Both T-fMRI and RS-fMRI clearly identify Broca and Wernicke regions. The 3DCNN method provides highly specific maps with large probability gradients at the margin of the language regions, as would be expected of a method trained on thousands of exemplars including millions of internal parameters. The T-fMRI experiment focused on expressive language and therefore emphasizes Broca's region. The 3DCNN map reflects the properties of spontaneous activity which characteristically is more symmetric than task responses. Robust delineation of both Broca's and Wernicke's area is not surprising as the 3DCNN was trained to recover the topography of T-fMRI responses in RS-fMRI data (14).

**Figure 2:**
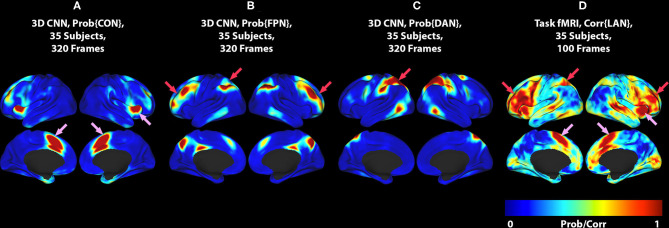
**(A)** 3DCNN map of the CON. **(B)** 3DCNN map of the FPN. **(C)** 3DCNN map of the DAN. **(D)** T-fMRI responses (reproduced from Figure 1C). Pink arrows point to components of the cingulo-opercular network (CON). Red arrows point to components of the dorsal attention network (DAN) and fronto-parietal control network (FPC). See text for discussion of these task-general functional systems.

The crucial difference between the two methods is that the word stem completion task activates areas not specifically associated with language (pink and red arrows in Figures 1, 2) in addition to areas that are specifically associated with language (pink arrows in Figures 1, 2). Task-general responses occur in the cingulo-opercular network (35), the dorsal attention system (23), and fronto-parietal control network (36). Additional discussion of these task-general systems is given below.

### Correspondence of Identified Language Maps With Neurosynth Reference

**Figure 3:**
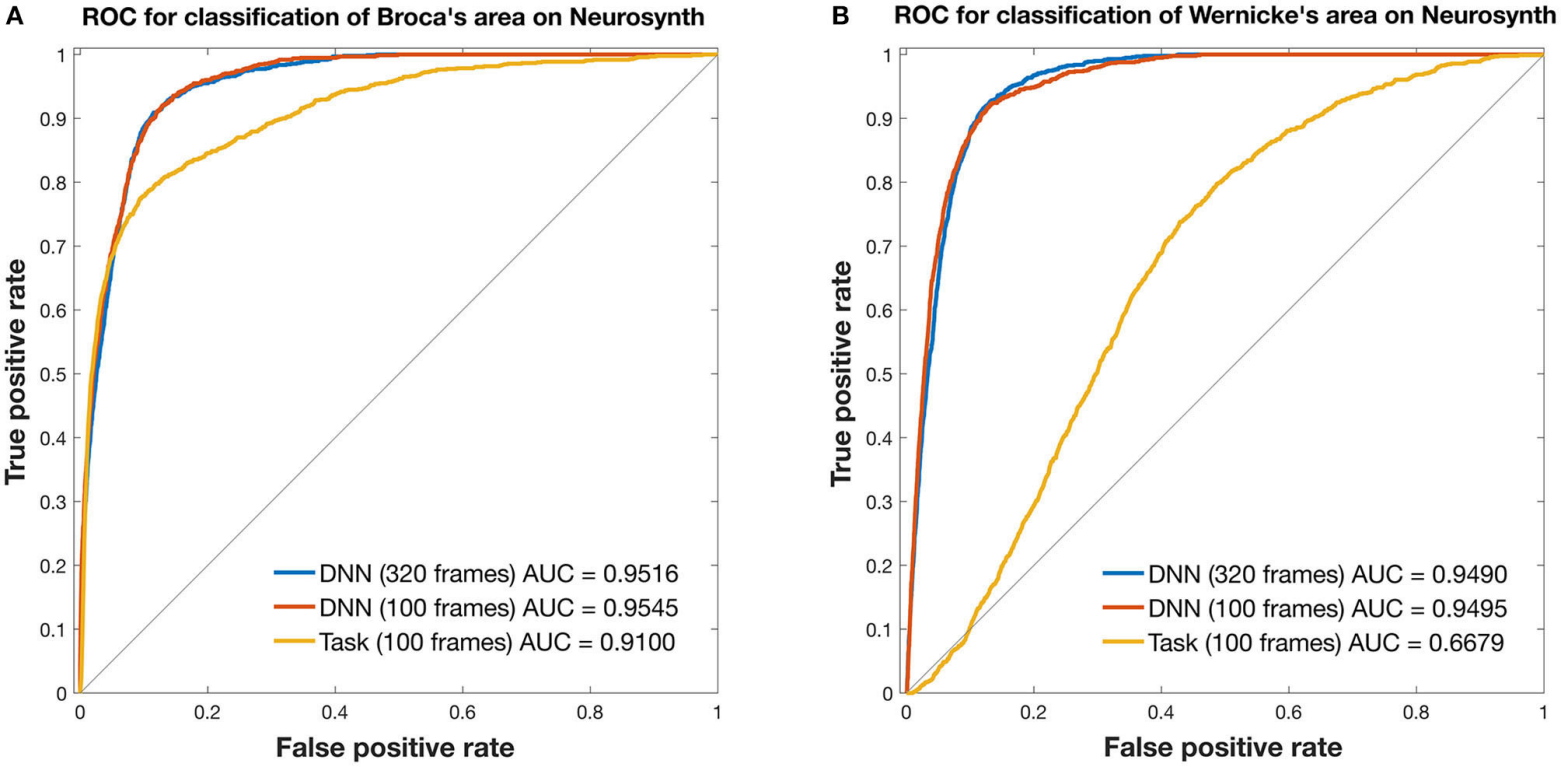
ROC curves for mapping the language network. Curves are from 3DCNN using 320 (blue) or 100 (red) resting-state fMRI frames. Additional curves are from 100 (yellow) T-fMRI frames. Ground truth labels are binary classes derived from thresholded Neurosynth data. All curves were constructed after averaging over 35 brain tumor patients. **(A)** Broca's area and **(B)** Wernicke's area are assessed separately. See text for details.

We assessed the topography of T-fMRI and RS-fMRI maps in relation to language ROIs defined on the basis of aggregated fMRI responses to language tasks (Figure 1D). ROC curves for Broca's area are presented in Figure 3A, and for Wernicke's area in Figure 3B.

In Broca's area, the 3DCNN AUC exceeded that for T-fMRI for both lengths of data, full length AUC = 0.9516 [0.9469, 0.9556] vs. 0.9100 [0.8992 0.9196] and for 100 frame data AUC = 0.9545 [0.9502, 0.9587] vs. 0.9100 [0.8992, 0.9196]. Notably, the AUC between the 3DCNN full length data and that for the shortened 100 frame data had overlapping 95% confidence intervals.

In Wernicke's area, the differences between the 3DCNN AUC and that of the T-fMRI was much larger, full length AUC = 0.9490 [0.9450, 0.9527] vs. 0.6679 [0.6549, 0.6811] and for 100 frame data AUC = 0.9495 [0.9449, 0.9537] vs. 0.6679 [0.6549, 0.6811]. As in the Broca's case, the AUC between the 3DCNN full length data and that for the shortened 100 frame data had overlapping 95% confidence intervals.

### Case Examples

This section demonstrates two case examples of the ability of the 3DCNN method to provide data acquired in individual patients and a comparison between the 3DCNN method and the T-fMRI at the individual level. Individual cases took approximately 4 h of computation time on a Dell (Austin, Texas) Power Edge 18 core with Nvidia (Santa Clara, California) v100 GPU.

### Case 1

**Figure 4:**
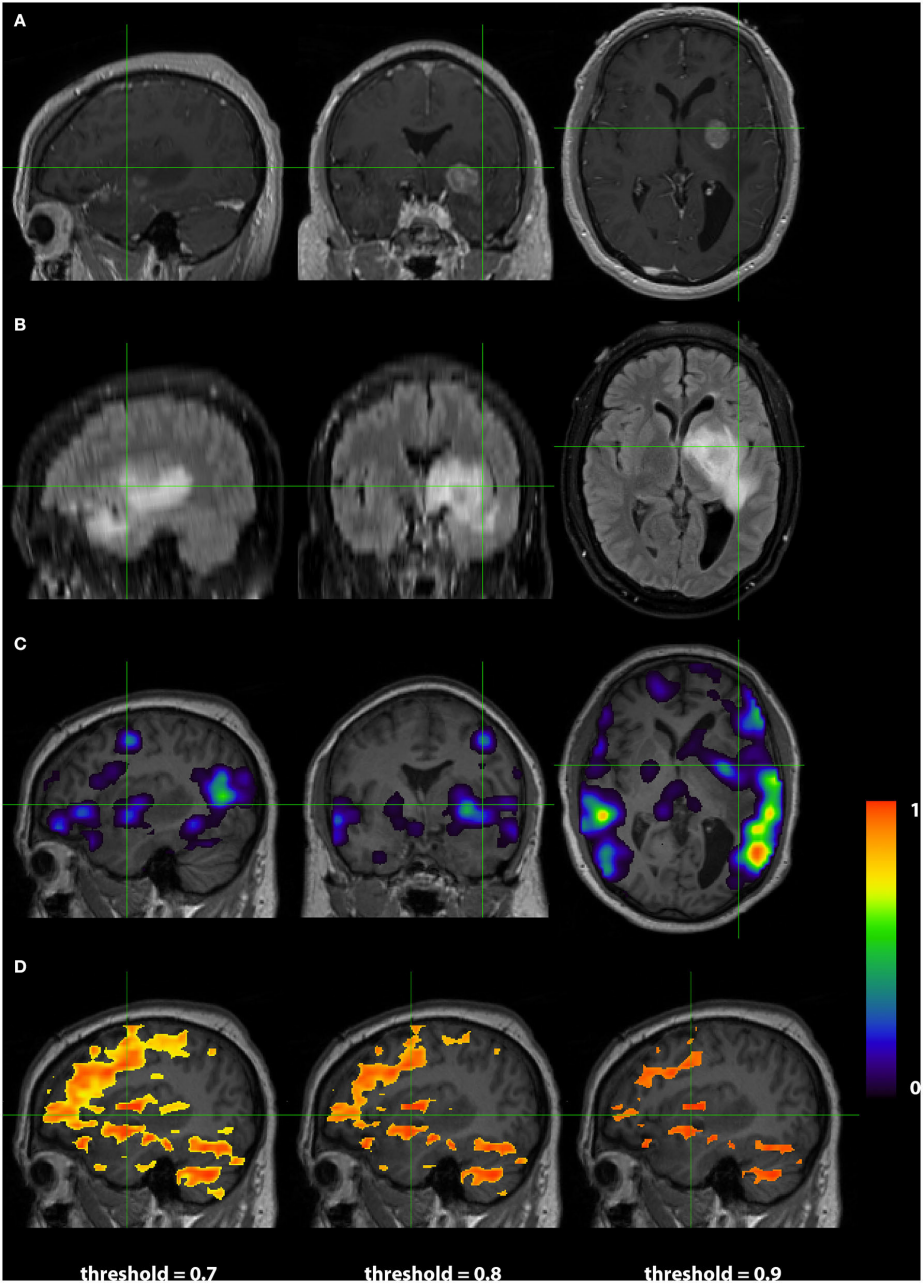
Exemplar patient RS003 with glioblastoma multiforme in the left basal ganglia region. **(A)** Contrast-enhanced T1 and **(B)** fluid-attenuated inversion recovery demonstrating the tumor and surrounding edema. **(C)** Probability map of language network from 3DCNN with significant overlap of the tumor and **(D)** probability map of language from the T-fMRI also showing overlap over the tumor. Deep learning results show probabilities > 0.02. Task fMRI thresholds are varied in accordance with clinical practice.

Images from a 44 year old right handed male (RS003) with glioblastoma multiform in the left basal ganglia region are presented in Figure 4. The top two rows display the anatomy with a post contrast T1-weighted and FLAIR images. The bottom two rows display the language localization information from the 3DCNN and T-fMRI overlying the anatomical images. We provide the T-fMRI at several thresholds in accordance with clinical practice. Although the T-fMRI appears noisier (bottom row) than the 3DCNN (third row), the information provided by both methods is similar with significant overlap of the localized language area with the tumor location.

### Case 2

**Figure 5:**
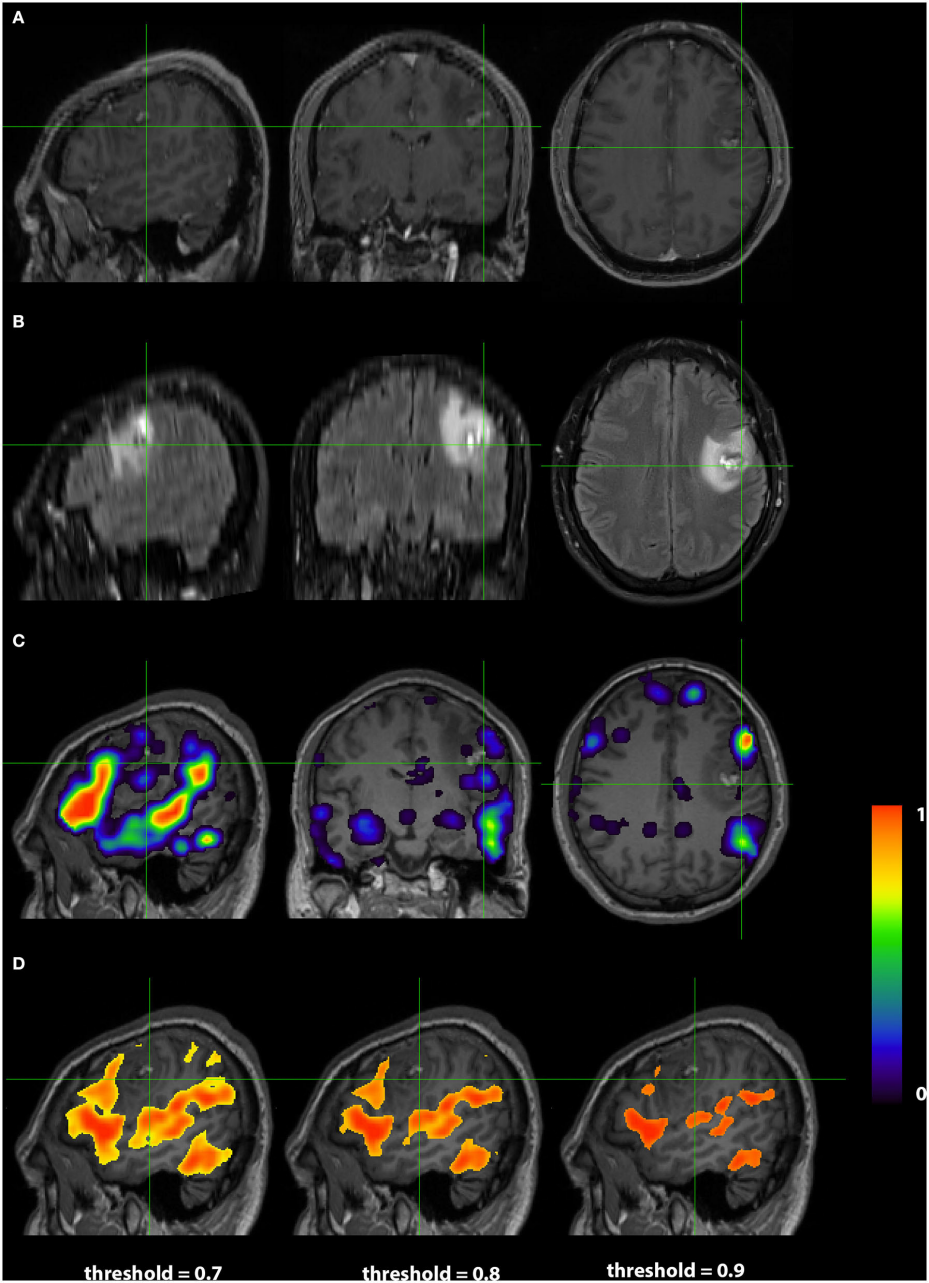
Exemplar patient RS004 with high grade glioma with anaplastic features in the subcortical left frontal lobe. **(A)** Contrast-enhanced T1 and **(B)** fluid-attenuated inversion recovery demonstrating the tumor and surrounding edema. **(C)** Probability map of language network from 3DCNN with mild overlap of the tumor and **(D)** probability map of language from the T-fMRI also showing mild overlap over the tumor. Deep learning results show probabilities > 0.02. Task fMRI thresholds are varied in accordance with clinical practice.

Images from a 24 years old right handed male (RS004) with anaplastic glioma in the subcortical left frontal lobe are presented in Figure 5. As in Case 1, The top two rows display the anatomy with a post contrast T1-weighted and FLAIR images. The bottom two rows display the language localization information from the 3DCNN and T-fMRI overlying the anatomical images. In this case, the sagittal views of the two methods are very similar, although the 3DCNN (third row) demonstrates more overlap of the language localization with the tumor as compared to the T-fMRI (bottom row).

## Discussion

The current work demonstrates that the representation of language in the brain can be identified using 3DCNN analysis of RS-fMRI data (Figure 1, white arrows). It should be noted that the 3DCNN was trained to identify language-associated parts of the brain using T-fMRI acquired at Washington University School of Medicine [same training set used by (14)]. The Neurosynth-derived map was obtained from an independent meta-analysis of 107 reported neuroimaging studies. Nevertheless, the 3DCNN and Neurosynth maps are strikingly similar.

The differences between T-fMRI and 3DCNN maps are instructive. The word-stem completion task activated the dorsal anterior cingulate (dACC) (a.k.a the rostral cingulate zone) as well as the right anterior insula (Figures 1, 2, pink arrows). These regions are components of the salience network (37), also known as core task-control regions (35, 38). The core task control system is recruited by a wide variety of goal-directed behaviors (38–40). Functions attributed to the dACC include task control (41, 42), error monitoring (43), and conflict detection (44). Additional T-fMRI responses not specific to the LAN occurred in the left superior parietal lobule and the left middle frontal gyrus. These regions are components of the dorsal attention network (DAN) and the fronto-parietal control network (FPC) (Figures 1, 2, red arrows). The DAN responds to any task requiring directed spatial attention (23, 45–47). The FPC supports goal-directed analysis of environmental stimuli (48–50). These functional systems are recruited by the word stem completion task as it requires directing attention to and analyzing stimuli presented on an electronic display.

The present results raise the possibility of distinguishing between parts of the brain that are language specific (28) vs. task-general (38–40). This distinction may be of value in selected neurosurgical cases. Although the DAN and dACC are not conventionally classified as “eloquent” (51), injury to these areas can lead to attentional deficits (52) and to loss of motivated behaviors (53), respectively.

An additional important observation evident in Figure 1 is that the localization of the language network using the 3DCNN appears remarkably tolerant to limited quantities of RS-fRMI data (Figures 1A,B). This characteristic could lead to reduced RS-fMRI acquisition times.

Figure 3 compares T-fMRI vs. 3DCNN as regards localization of Broca and Wernicke areas as defined *a priori*, according to a large collection of T-fMRI studies aggregated by Neurosynth (17). According to the AUC measure, 3DCNN had a small but significant advantage over T-fMRI in localizing Broca's area (Figure 3A). The difference was much larger in Wernicke's area (Figure 3B). This result is understandable as the word stem completion task is an expressive language task that preferentially activates Broca's area. Performing several different T-fMRI studies better characterizes the language system (54–56). Figure 3 also demonstrates 3DCNN tolerance to a limited quantity of data: there is no significant difference in AUC corresponding to 100 vs. 320 frames (red and blue curves).

The case examples demonstrate 3DCNN functional mapping in individual patients with brain tumors (Figures 4, 5). The higher specificity and sharper margins of the 3DCNN method in comparison to T-fRMI is promising. A prospective comparison of the 3DCNN RS-fRMI method vs. T-fMRI remains to be done.

Limitations of our study include first, that it is formulated in terms of group-level analyses; hence, our results do not directly speak to the question of which technique provides the best functional localization of language in individuals. Our focus has been on regions instantiating expressive and receptive language functions (Broca and Wernicke). Further study will be needed to evaluate the utility of classifier-based analysis or RS-fMRI for localizing functions such as reading, articulation, and prosody. Additionally, we make no claims for the 3DCNN in determining language lateralization. As our data are retrospective, we have no means of assessing the potential effects of neuro-vascular uncoupling (57). Similarly, we have not considered how anatomical distortion owing to tumor mass may have affected our results, although the average tumor size was small (~44 mL). This work omits comparison with intraoperative brain mapping; that comparison is reported in prior related work (58). The relatively poor performance of T-fMRI in localizing Wernicke's area is a limitation of the word stem completion task. This limitation could be overcome by use of a wider range of language tasks (33, 56). Distorted brain anatomy in tumor patients compromises affine atlas registration; however, this issue will have affected T-fMRI and 3DCNN equally. Finally, a definitive comparison of RS-fMRI vs. 3DCNN in terms of patient outcomes would require a prospective, multi-center, clinical trial (59).

## Conclusion

This study demonstrates that 3DCNN analysis of RS-fMRI data is able to accurately and specifically localize the language network in patients with brain tumors. In addition to the inherent advantages of RS-fMRI, specifically, limited requirement for patient cooperation, the 3DCNN method provides robust results with limited quantities of data, which is an advantage in the clinical setting. We anticipate that this method will lead to improved pre-surgical localization in future applications.

## Data Availability Statement

The datasets generated for this study are available on request to the corresponding author.

## Ethics Statement

The studies involving human participants were reviewed and approved by Washington University Human Research Protection Office. The patients/participants provided their written informed consent to participate in this study.

## Author Contributions

JS, PL, JL, KP, and CH: conceptualization and methodology. DD, KP, JL, PL, and AD: data curation. JL, PL, KP, CH, and BS: formal analysis. EL and JS: funding acquisition. EL, JS, JL, and PL: investigation. EL, JS, and BA: project administration, supervision, and resources. PL, JL, KP, CH, and AS: software. PL and JL: validation. DD, JL, and BS: visualization. JS, AS, and KP: writing original draft. AS, JS, BS, JL, PL, EL, BA, DD, AD, and CH: writing review/editing. All authors contributed to the article and approved the submitted version.

## Conflict of Interest

The authors declare that the research was conducted in the absence of any commercial or financial relationships that could be construed as a potential conflict of interest.
